# Genome-wide mapping of G-quadruplex DNA: a step-by-step guide to select the most effective method

**DOI:** 10.1039/d4cb00023d

**Published:** 2024-03-25

**Authors:** Silvia Galli, Gem Flint, Lucie Růžičková, Marco Di Antonio

**Affiliations:** a Imperial College London, Chemistry Department, Molecular Science Research Hub 82 Wood Lane London UK m.di-antonio@imperial.ac.uk; b Institute of Chemical Biology, Molecular Science Research Hub 82 Wood Lane London UK; c The Francis Crick Institute 1 Midland Road London UK

## Abstract

The development of methods that enabled genome-wide mapping of DNA G-quadruplex structures in chromatin has played a critical role in providing evidence to support the formation of these structures in living cells. Over the past decade, a variety of methods aimed at mapping G-quadruplexes have been reported in the literature. In this critical review, we have sought to provide a technical overview on the relative strengths and weaknesses of the genomics approaches currently available, offering step-by-step guidance to assessing experimental needs and selecting the most appropriate method to achieve effective genome-wide mapping of DNA G-quadruplexes.

## Abstract 2

Overview of the strengths and weaknesses of the current genomics approaches to mapping DNA G-quadruplex structures, facilitating the selection of the most suitable method based on specific needs.

## Graphical abstract

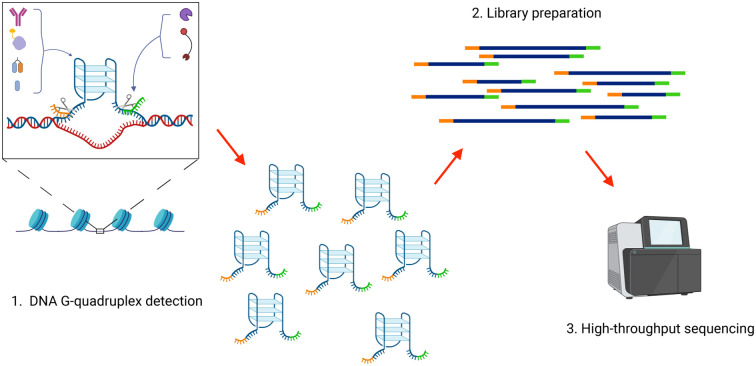

## Introduction

### DNA G-quadruplexes

**Fig. 1:**
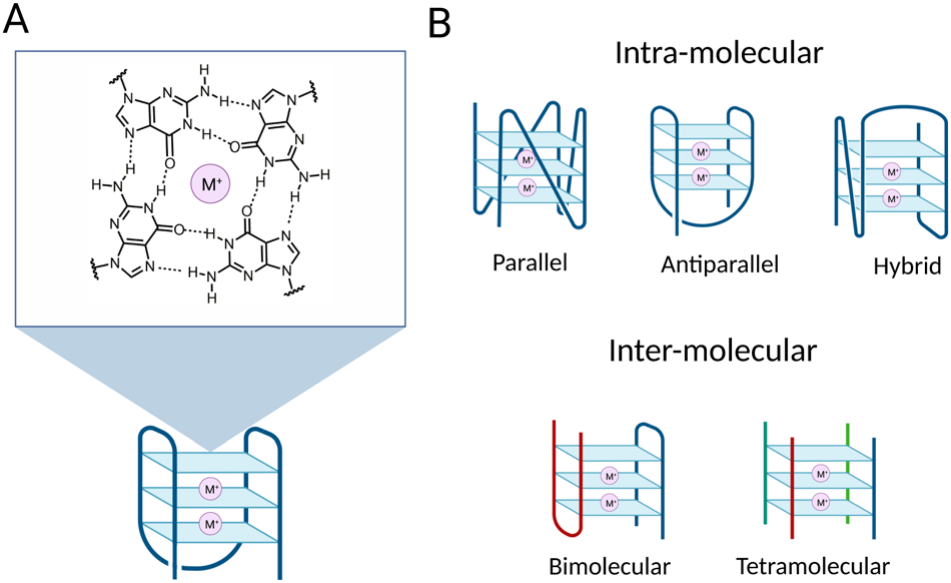
Schematic of a G-quadruplex structure (G4). (A) Example of a three-tetrad G-quadruplex structure with schematic of a G-tetrad composed of four co-planar guanines. The O_6_ and N_7_ of a guanine established hydrogen bonds with N_1_ and N_2_ of an opposite guanine to form a G-tetrad. The structure is stabilised by a central monovalent cation (M^+^) coordinating O_6_ atoms of the four guanines. G4 stabilisation by metal coordination increases following the K^+^ > Na^+^ > >Li^+^. (B) Schematic of different G4 topologies. Top: Intra-molecular G4s, classified according to the orientation of the strands constituting the G4-structure. Bottom: Inter-molecular G4 structures: bimolecular G4 structure on the left and tetramolecular G4 structure on the right. Strands from different molecules composing the G4 structures are in different colours.

G-quadruplex structures (G4s) are DNA secondary structures that arise from guanine-rich single-stranded DNA. More specifically, G4s are formed when Hoogsteen hydrogen bonding occurs between four guanines to associate in a planar structure known as a G-tetrad (Fig. 1(A)).^1^ Two or more tetrads can then self-assemble by means of π–π stacking interactions to form a full G4-structure (Fig. 1). The formation and subsequent stabilisation of the structure is promoted by alkali monovalent cations that coordinate to the guanine oxygens, primarily K^+^ ions.^2,3^ G4s can be classified into distinct topologies based on the relative orientation of the DNA strands composing the structure, leading to parallel, antiparallel or hybrid G4s (Fig. 1(B)).^4^ Additionally, G4s can be classified as intra-molecular or inter-molecular, contingent on whether the structure is formed by an individual or multiple DNA strands (Fig. 1(B)).^5^

Although *in vitro* formation of G4s was first described more than 60 years ago, it is only in the last decade that their formation within cells and their potential to regulate biological processes has been unraveled.^6,7^ Indeed, it has been widely speculated that G4s could be biologically functional given the high abundance of G-rich sequences at functional genomic sites. However, without the tools to map and visualise these structures on a genome-wide scale, it has been impossible to investigate the biology associated with G4-formation in detail. To overcome this, one of the first steps to gain insight into the potential biological relevance of G4-structures was to predict the location of these structures within the genome. Many *in silico* and experimental studies have been performed and described over the years to achieve genome-wide mapping of G4-structures. Altogether, these studies revealed that G4s are particularly enriched at regulatory regions, for instance at promoters, enhancers, 5′ UTRs, 3′ UTRs and splicing sites,^8,9^ supporting the hypothesis that G4s might play a role in the modulation of biological processes, like transcription. Moreover, their enrichment at oncogene promoters suggested that they could be an attractive target for cancer intervention.^10^ More recently, the potential of G4s in supporting long-range chromatin looping interactions has also been suggested, which further strengthens the idea of G4s acting as key regulatory elements in gene expression.^11,12^

### Computational prediction and G4-mapping *in vitro*

Several algorithms looking at specific sequence features have been developed to computationally predict the genomic distribution and prevalence of G4-structures. One of the first algorithms developed was Quadparser, which uses a canonical G4-folding sequence, the motif d(G_3+_N_1–7_G_3+_N_1–7_G_3+_N_1–7_G_3+_), to identify regions that are likely to form G4s under physiological conditions.^13^ Recently, more advanced algorithms were designed to reduce false positives and negatives generated with Quadparser. One recent example is G4Hunter,^14^ which was designed to assess G-richness of a sequence, and G-skewness between the complementary strands to provide a more holistic propensity score of the likelihood of the sequence to fold into a G4. From this, the sequences predicted to form stable G4s were further validated *in vitro* using standard biophysical assays (*i.e.* Circular Dichroism) to confirm G4 formation under physiological conditions. More recently, artificial intelligence and machine learning strategies have also been deployed to identify putative G4-folding sequences in a high throughput fashion, such as Penguinn, G4detector, Quadron and G4mismatch.^15–18^ These strategies rely on training these algorithms by training them on experimental data (biophysical and genomics) rather than looking solely at sequence features, which makes their effectiveness highly dependent on the depth and the quality of the data available.

Computational methods have been extremely powerful in generating the very first reference maps of putative G4-folding sequences across the human genome. However, these methods fail to account for the dynamic chromatin landscape of a living cell and for the transient nature of G4-structures, which makes them not sufficiently comprehensive to fully assess the biological relevance of G4-structures. Consequently, methods to experimentally map endogenous G4s in chromatin are essential to validate G4-formation in the cellular context and to enable investigation of G4-biology.

The first experimental high-resolution whole-genome G4-map was reported in 2015 with the development of the sequencing method G4-Seq.^19^ This experimental strategy combines high-throughput sequencing with the polymerase stalling abilities of G4-structures, which causes errors in base calling and sequencing readouts that can be used as indicators of G4 formation. This method was instrumental for the development of genomic mapping of G4 structures and identified ∼700 000 sequences potentially folding into experimentally observed G4s (OQs) in the genome, over 450 000 more than what was initially indicated computationally.^13^ Of note, experimental mapping allowed the characterisation of several non-canonical, looping, and bulged G4 structures, besides those comprised of only two tetrads, which are more difficult to predict *in silico* using primary sequence alone.^19,20^ A limitation of this technique is the use of isolated DNA, which does not reflect the dynamic nature of chromatin that exists in the cellular context and that could strongly affect G4 formation and distribution in cells.

### Probes compatible with chromatin mapping and visualisation of G4s

To achieve genome-wide mapping of G4-structures in chromatin, many small molecules and antibodies have been developed and validated over the past couple of decades.^21–25^ More than 1000 G4-binding ligands have been developed to date, all exhibiting high specificity for G4s over double-stranded DNA. One of the most widely used G4-ligand is pyridostatin (PDS), firstly described in 2008.^26^ The first studies using PDS in cells showed that the stabilisation of G4s by this small-molecule triggers a DNA damage response, causing cell cycle arrest and replication and transcription-dependent double-strand breaks, which have been mapped to indirectly detect G4-folding sequences in cells for the first time.^21^ Later, PDS analogues were employed in imaging techniques to visualise G4s in human cells. This includes real-time G4 detection in living cells under non-perturbative conditions through single-molecule imaging using the PDS analogue SiR-PyPDS.^27^ This method highlighted the dynamic folding and unfolding nature of G4 structures within the cell, confirming its dependency on active transcription and replication.

Additionally, G4-selective antibodies have been generated such as Sty49 and hf2 to visualise G4s using immunofluorescence on ciliates and to achieve immuno-precipitation from isolated human DNA, respectively.^25,28^ However, the G4-selective scFv antibody, BG4, firstly reported in 2013, was the first antibody successfully used for direct chromatin immuno-precipitation (ChIP) of G4-structures.^25,29^ BG4 binds with low nanomolar affinity to a broad range of G4s, independently of their topology, and was initially used to visualise G4s in mammalian cells through immunofluorescence.^25,30^ Despite their use for G4-visualisation with microscopy, both BG4 and PDS-based probes have been also used to attain G4-mapping in human chromatin. In this review, we aim to critically assess the current methods developed to map DNA G4-structures in human cells and provide direct guidance on choosing the best method to employ based on experimental needs and relative strengths of individual methods. We will compare and contrast the limitations of different methods and highlight the importance of cross-validation of the generated maps by using a combination of the different genomics methods available.

## Current approaches to G-quadruplex genome-wide mapping

**Fig. 2:**
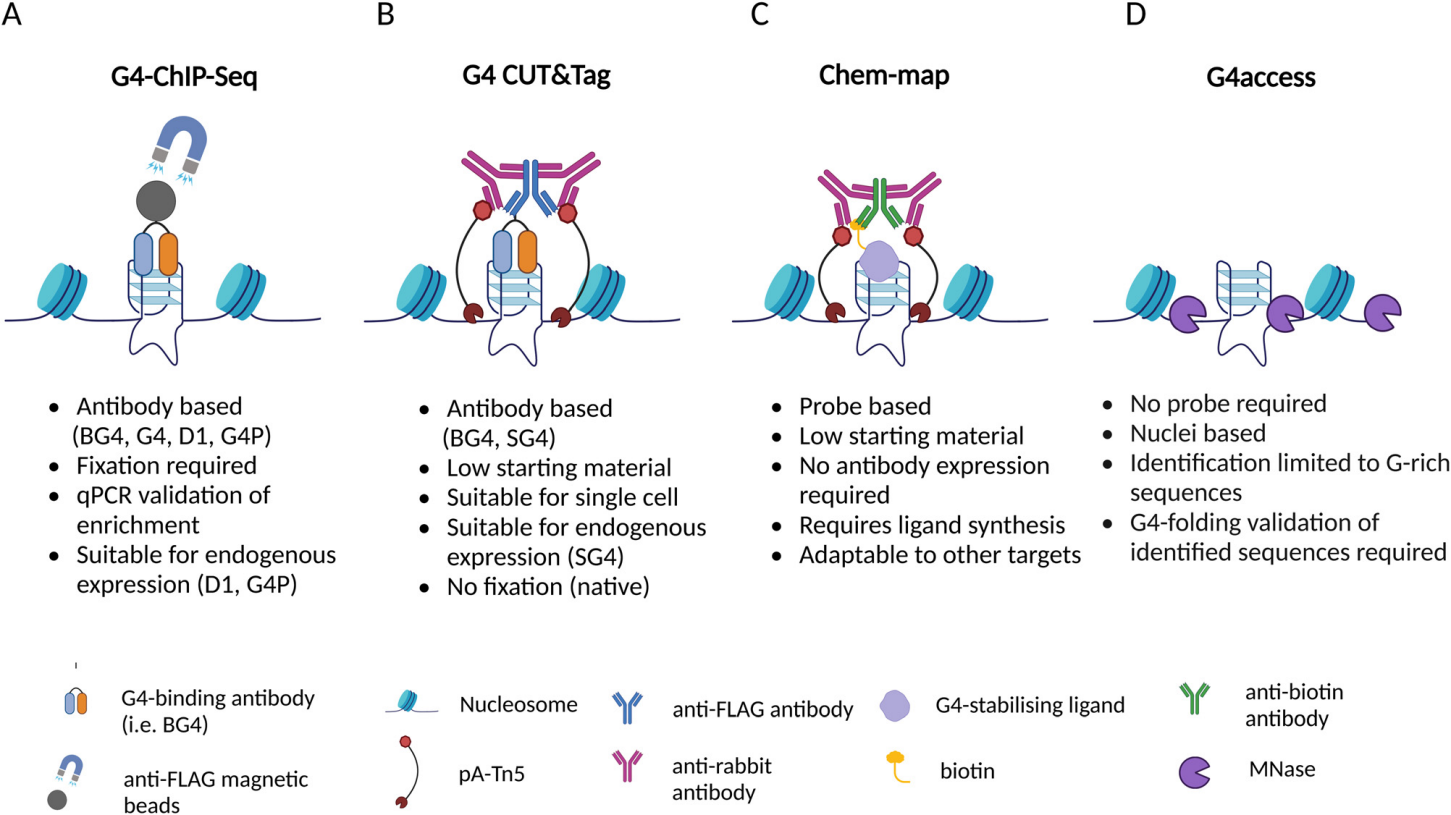
Summary of the techniques currently available to map G4s in the human genome and brief overview of their features. (A) Schematic of G4 ChIP-Seq. The illustration represents a region of the genome forming a G4 structure. The G4-specific antibody, tagged with a 3xFLAG, is targeted by magnetic beads coated with anti-FLAG antibody. (B) Schematic of G4 CUT&Tag. The G4 structure is targeted by a G4-specific antibody tagged with a 3xFLAG; the tag is recognised by an anti-FLAG antibody, targeted by a tertiary IgG antibody, which recruits protein A-Tn5 complex (pA-Tn5) that cleave DNA at the surrounding sites. (C) Schematic of Chem-map. A G4 structure is bound by a biotinylated G4-ligand; the complex is recognised by an anti-biotin antibody, subsequently targeted by a secondary IgG antibody that recruits the pA-Tn5 complex at the site to cleave the DNA surrounding the G4 structure. (D) Schematic of G4access. MNase cleaves DNA in open chromatin regions before G-rich stretches, including sequences folded into G4s.

Many experimental strategies are currently available to underpin the location of G-quadruplexes (G4s) throughout the human genome. All these methods are based on the isolation of chromatin fragments containing G4s followed by high-throughput sequencing, which allows the detection of individual G4s in the genome by aligning the sequencing reads against a reference genome. Although this is a shared commonality of all the experimental methods used to map G4s genome wide, there are profound technical differences amongst the approaches available and described to date (Fig. 2).

Generally speaking, G4-mapping strategies can differ quite substantially from each other by being probe-based (ChIP-Seq, CUT&Tag, Chem-map) or probe-independent (G4access), as well as by requiring chemical fixation (ChIP-Seq) or not (CUT&Tag, Chem-map, G4access). In addition, some methods might be more compatible to analyse limited amounts of chromatin samples (CUT&Tag, Chem-map). In the following sections, we will describe in detail the technicalities associated with each one of the available strategies and highlight the benefits and limitations associated.

## Mapping G4s with antibodies or artificial proteins

### ChIP-Seq

The generation of G4-binding antibodies offered a ground-breaking opportunity for the identification of the genomic location of G4s in chromatin.^22–25^ Antibodies targeting DNA-binding proteins are often used in chromatin immunoprecipitation followed by high-throughput sequencing (ChIP-Seq) to map their binding sites across the genome. Therefore, similar approaches have been adapted to a DNA-binding antibody in order to map DNA G4s by immunoprecipitation. To date, G4-binding antibodies and an engineered protein have been successfully used for genome-wide mapping of G4s by ChIP-Seq.

**Fig. 3:**
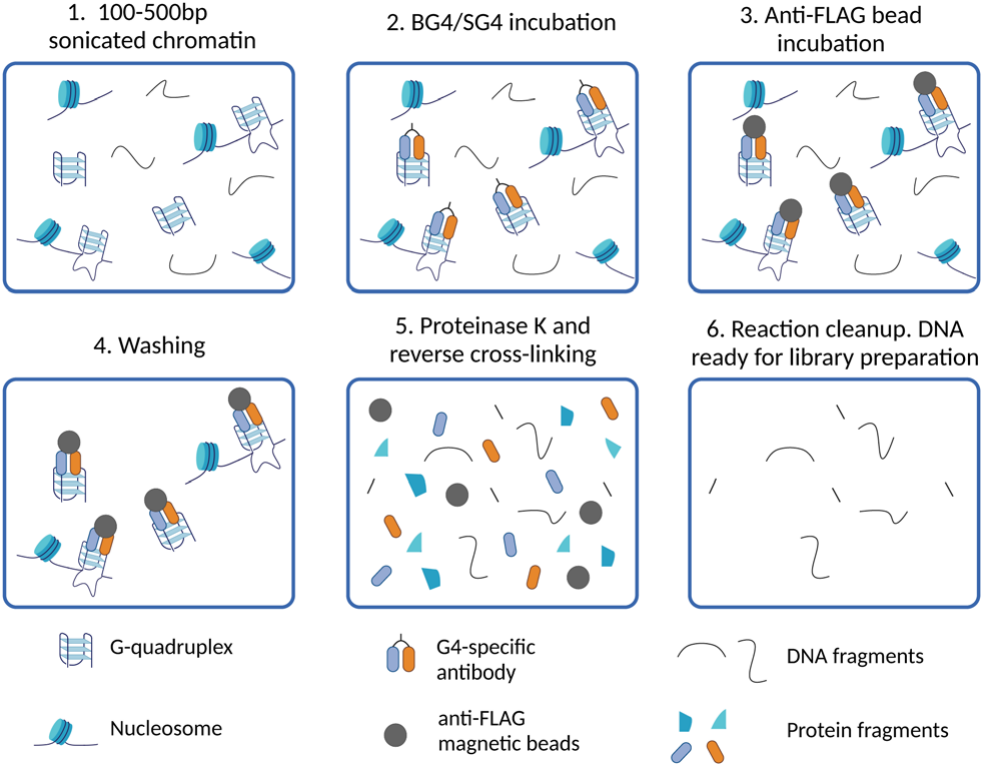
Schematic of G4 ChIP-Seq workflow. (1) After cell harvesting, chromatin is cross-linked and extracted from isolated nuclei, then sonicated to obtain DNA fragments of 100–500 bp; (2) cross-linked chromatin fragments are incubated with a G4-specific antibody (BG4 or SG4) to immuno-precipitate G4-containing fragments; (3) anti-FLAG beads are used to isolate fragments containing G4s by binding to the 3xFLAG tag fused BG4 or SG4; (4) washing steps are performed to reduce non-specific interactions with fragments that do not contain G4s; (5) reverse cross-linking is performed to obtain free DNA by heating and enzymatic digestion of proteins with proteinase K; (6) DNA fragments are eluted and barcoded libraries for high-throughput sequencing are prepared.

**Fig. 4:**
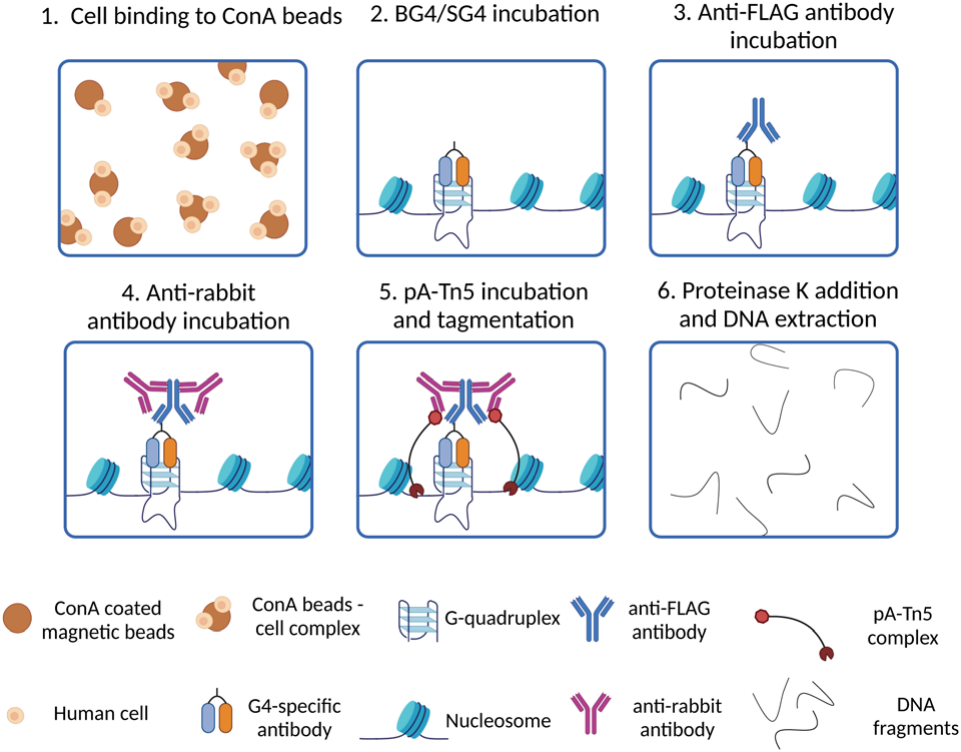
Schematic of G4 CUT&Tag workflow. (1) Native cells/nuclei are immobilised to ConA beads; (2) cells/nuclei-ConA beads complexes are incubated with BG4 or SG4 to target genomic G4s; (3) a rabbit anti-FLAG antibody is used to target the 3xFLAG present on BG4 or SG4 antibodies; (4) the samples are incubated with an anti-rabbit antibody to target the rabbit anti-FLAG antibody and recruit pA-Tn5 to the G4 sites; (5) pA-Tn5 digests DNA surrounding the G4 sites and inserts adaptor for preparation of high-throughput sequencing libraries; (6) all the proteins present in the samples are digested by proteinase K treatment, then DNA extracted and libraries for high-throughput sequencing are prepared by PCR amplification with barcoded primers.

**Fig. 5:**
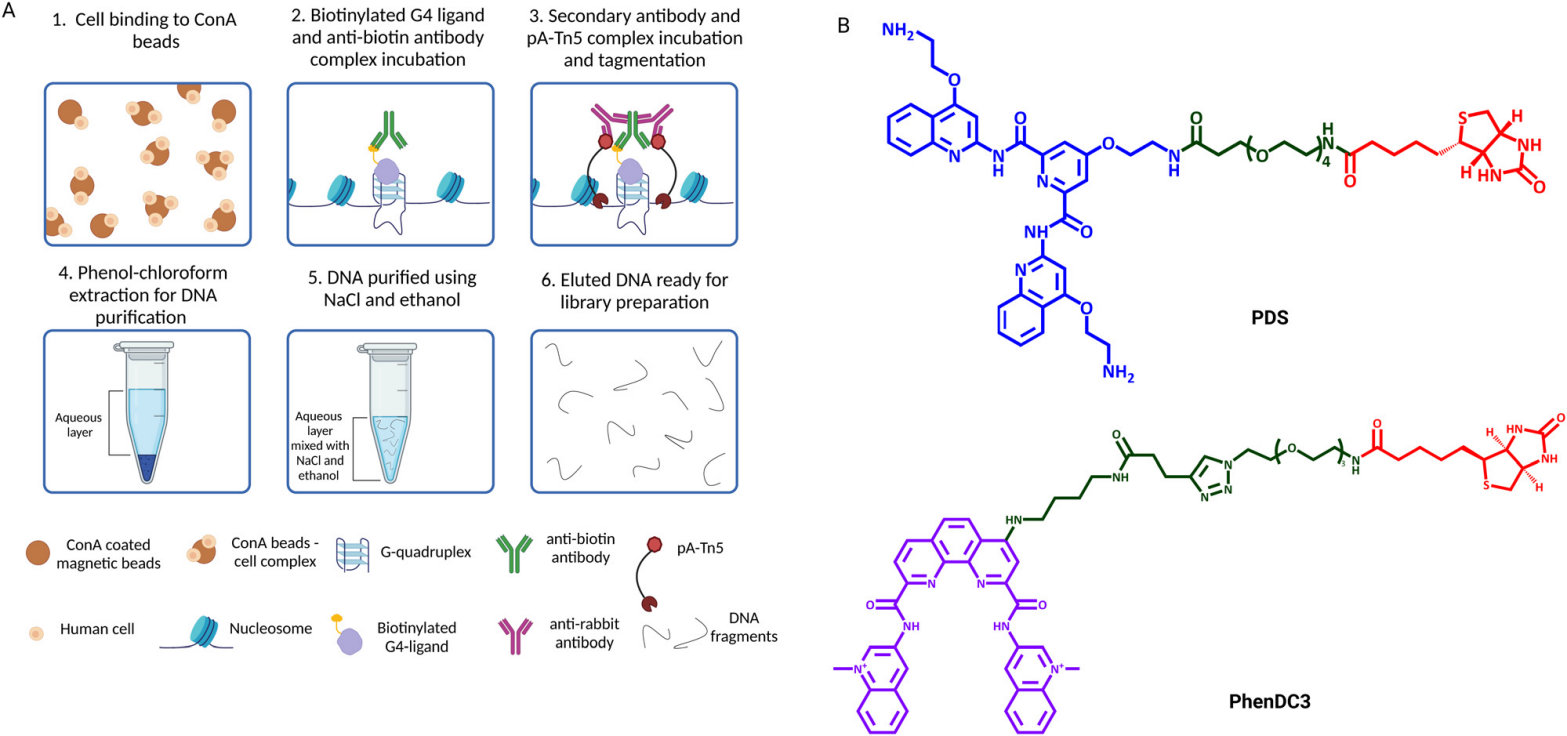
(A) Schematic of Chem-map workflow. (1) Native cells or nuclei are immobilised to ConA beads; (2) cells/nuclei-ConA beads complexes are incubated with biotinylated G4 ligands, which are subsequentially targeted by a rabbit anti-biotin antibody; (3) an anti-rabbit antibody is added to recruit pA-Tn5 to the G4 sites, which tagments DNA surrounding the G4 sites; (4) and (5) DNA is extracted from cells through aqueous phase separation by phenol chloroform, followed by high-salt (NaCl)/ethanol DNA precipitation; (6) DNA is eluted and libraries for high-throughput sequencing are prepared by PCR amplification with barcoded primers. (B) Chemical structures of PDS and PhenDC3 biotinylated ligands used in Chem-map.

**Fig. 6:**
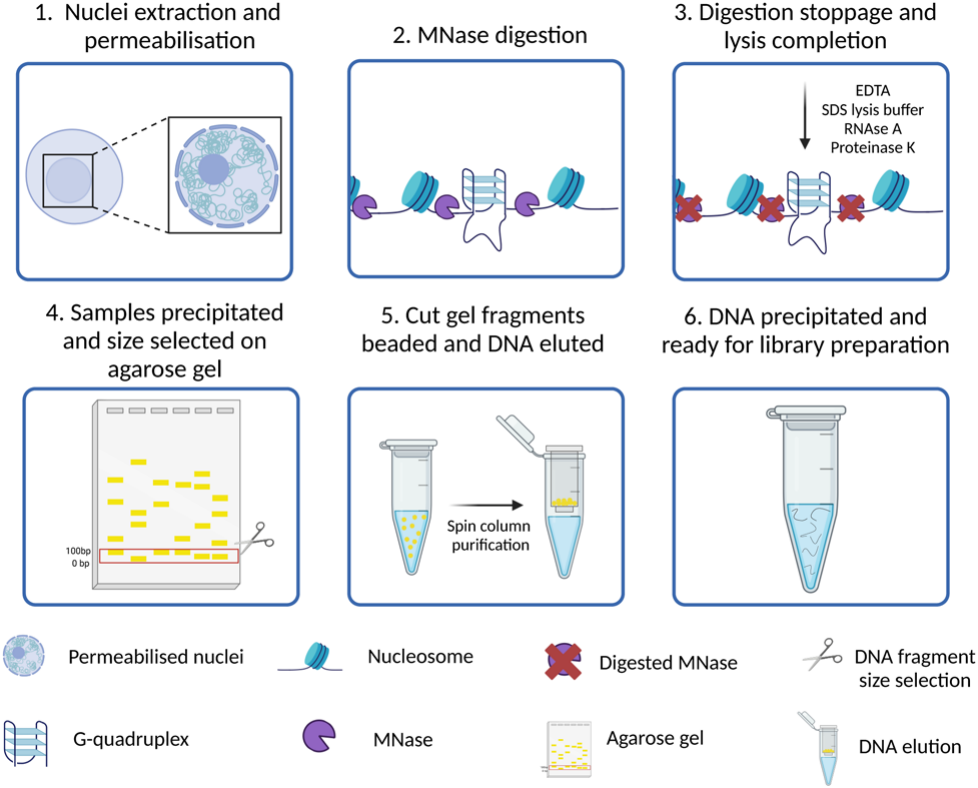
Schematic of G4access workflow. (1) Nuclei are isolated from native cells using a hypertonic buffer; (2) the nuclease MNase digests open chromatin sites in proximity of DNA G-rich stretches; (3) cold temperatures and SDS are used for DNA extraction; (4) the purified DNA fragments are size selected and extracted from agarose gel; (5) and (6) eluted DNA is used to prepare libraries for high-throughput sequencing.

The first G4 ChIP-Seq protocol was reported by the Balasubramanian group in 2016 and relied on the use of the G4-selective antibody BG4.^9,25^ This experiment enabled direct detection of endogenous DNA G4s in human chromatin for the first time, which followed closely the first indirect genome-wide map of G4s obtained by ChIP-Seq of the DNA damage marker γH2AX generated upon treatment with PDS.^21^ More recently, the G4-ChIP protocol has also been adapted for the use of a nanobody (SG4).^24^ The G4-ChIP protocol is schematically illustrated in Fig. 3. In essence, chromatin is cross-linked by formaldehyde fixation, followed by sonication to obtain DNA fragments of ∼100–500 bp (Fig. 3-(1)).^31^ The need of fixation prior to fragmentation is key to preserve essential features of chromatin architecture, including histones and accessible sites. Both BG4 and SG4 are fused to a FLAG-tag and are used as recombinant antibodies to bind and enrich chromatin fragments containing G4s by immuno-precipitation (Fig. 3-(2)). Such fragments are precipitated using magnetic beads decorated with an anti-FLAG antibody and unbound fragments are washed off (Fig. 3-(3) and (4)). After reverse cross-linking by heating and proteinase K treatment for protein digestion (Fig. 3-(5)), the precipitated DNA fragments are sequenced using standard Illumina sequencing and aligned back to the genome of reference (Fig. 3-(6)). It is highly recommended to assess the quality of G4-ChIP enrichment prior to sequencing using qPCR. This is to ensure that the antibody immuno-precipitation step has successfully enriched for sequences of the genome bearing a G4-structure. To achieve this, part of the eluted ChIP fraction is amplified using primers targeting a region of the genome that is known to form a G4 (G4-positive region). A G4-negative region, which does not fold into a G4, is included to establish the background of immunoprecipitation. A fraction of fragmented DNA that was not subjected to BG4 precipitation, known as input sample, is also included in the assessment for normalisation purposes. There are two key factors to consider in assessing the quality of G4-ChIP: (1) the amount of DNA precipitated by the G4-antibody relative to the unprecipitated input sample (% input) and (2) the relative enrichment of DNA recovered from G4-positive regions over G4-negative regions (G4-fold enrichment). A G4-ChIP library can be considered of sufficient quality for sequencing when the % Input in G4-positive regions is >5% and when the fold enrichment over G4-negative regions is 5-fold or more. While this is a powerful quality control step, its main limitation is the requirement of knowing in advance the G-rich sequences that are folded into a G4 in the chromatin context. These positive control regions are reasonably well established in the human genome, but this could pose challenges when G4-ChIP is performed for the first time on other organisms in which the genomic location of G4s is still uncharacterised.

To date, BG4 has been used in ChIP-Seq to map G4-prevalence in a variety of human cell lines, including cancer cell lines such K562, IMR90, HaCaT, U2OS, 93T449 (WDLPS), and 293T,^9,31–34^ and other cell types including human embryonic stem cells and human keratinocyte (NHEK).^29,35^ The number of G4 sites detected using BG4 ChIP-Seq spans from ∼1000 to ∼20 000, depending on the cell line considered. More recently, G4-ChIP has been performed using a different antibody. More specifically, G4s were mapped in the genome of sporadic Alzheimer's disease (AD) neurons by employing the IgG 1H6 antibody.^23,36^ Although 1H6-based G4-ChIP was performed using a kit and following a protocol optimised for histones that was not further adapted for G4s, it shares the same principles of cross-linking of the chromatin and fragmentation by sonication, followed by precipitation of fragmented chromatin by recombinant 1H6 and a final DNA elution step.

Using G4 ChIP, it has been noted that a great variation in terms of total number of G4s detected can be observed when comparing cancer cells against non-cancerous cells, and that G4-prevalence is particularly high at promoters of oncogenes that are highly expressed in cancer cells.^9^ Moreover, a significant decrease in the number of G4s occurs during differentiation of human embryonic stem cell (hESC, ∼18 000 G4 sites) to downstream lineages (NSC, CNCC with ∼4000 and ∼9000 G4 sites, respectively).^35^ Upon differentiation, many genes either lose or acquire a G4 at their promoters. The expression of genes that acquired a G4 in their promoter increases, while loss of a promoter G4 often correlates with decreased gene expression. Many genes that lose a G4 during differentiation are important for a pluripotent phenotype, whilst genes that gain a G4 are usually cell lineage specific. This is also correlated with an increase or decrease in chromatin accessibility in the case of gain or loss of a promoter G4 respectively. Overall, these studies collectively suggested that G4s might play a role in the regulation of gene expression and might act as epigenetic marks, both in disease state like cancer but also in fundamental cellular differentiation. Similarly, the nanobody SG4 has been recently developed and used to achieve G4 ChIP-Seq in K562 and U2OS, leading to the detection of around 10 000 and 20 000 G4 sites, respectively, which is consistent with what was observed with BG4 ChIP-Seq.^24^ Conversely, the 1H6 ChIP-Seq identified ∼1400 G4s in neurons.^36^ The substantial difference in the number of G4s found by 1H6 compared to BG4 or SG4 could be down to the specific ChIP protocols, or the cell type used, as well as to the different G4-binding abilities of these probes. Indeed, the antibody 1H6 has been shown to have a strong preference for targeting G4s flanked by a T-rich stretch, which might lead to an overall underestimation of the total G4s present.^23^

An advantage of using SG4 is its undemanding production of active protein from bacterial cells. Indeed, it is possible to obtain ∼0.7 mg of nanobody from 100 mL bacterial culture, which is ∼30 times higher than an average BG4 production yield.^24^ This is probably due to its smaller size (∼18 kDa) compared to BG4 (∼31 kDa), and to the fact that SG4 is composed of a single domain. Conversely, BG4 is a heterodimeric antibody, which renders protein folding more challenging to achieve. Moreover, a mutant version of SG4, known as mSG4 R105A, is also available and useful to employ as a negative control in ChIP. The antibody mSG4 shows strongly reduced affinity for the G4s tested *in vitro* and fails to precipitate G4 fragments in ChIP.^24^ Therefore, this mutant could be used as a negative control in ChIP experiments and ChIP-qPCR assessment, which could be particularly valuable when assessing G4-prevalence in new organisms that do not offer validated positive control sites for qPCR. Additionally, SG4 has been demonstrated to function as a recombinant antibody as well as to be active upon endogenous expression in human cells, which offers alternative experimental strategies, as described in the next section.

### Endogenous expression of G4-binding probes for G4 profiling

The use of recombinant antibodies on extracted chromatin has some major limitations. The step of chromatin preparation, including cross-linking, extraction, and sonication, might affect the endogenous folding of G4 structures, although direct evidence to demonstrate this is yet to be presented. Additionally, because of the crosslinking step required in G4 ChIP-Seq, it is only possible to detect G4s that are not cross-linked to interacting proteins, which may hide some key endogenous G4 sites. For this reason, the alternative of directly expressing G4s probes in human cells has been investigated. When G4s probes are endogenously expressed in cells, they can directly interact with endogenous G4s prior to cross-linking, removing any bias associated with the fixation step. On the other hand, it is important to consider the possibility of promoting non-endogenous G4-formation upon overexpression of a G4-binding probe in cells, which is typically required for these experiments. Moreover, overexpression of an endogenous G4-probe may affect normal cell homeostasis by displacing endogenous G4-interacting proteins, which could both lead to false positive and negative G4-detection. This highlights how, in the attempt of overcoming some limitations imposed by a method, new confounding factors might be introduced, emphasising the need of combining multiple genome-wide strategies to attain a reliable overview of G4-prevalence in a given cell.

From a more technical standpoint, endogenous expression of G4-binding probes includes the transfection of cells with a vector compatible with human cells, containing the sequence of the desired probe and an antibiotic resistance cassette. This allows for antibiotic selection of the cells that can express the G4-binding probe. The expression could be either transient or stable. The latter involves the integration of the G4-probe sequence in the genome of the cell of interest, which requires around a month to be stably generated, while transient transfection is relatively quick and can be performed over a few days. The efficiency of cell transfection is variable and normally spans from 40% to 80%, which implies that a significant loss in cell number is expected in the process. Thus, this approach is preferable only when there is a large availability of cells that are relatively fast at replicating. For instance, this excludes samples derived from patients to study the impact of G4s in diseases, for which the collection of small samples for diagnosis is preferred. As this approach requires a high amount of starting material and is often time-consuming, in many cases, it is more advantageous to employ a recombinant antibody in ChIP-Seq, as discussed in the previous section, or in CUT&Tag, described in the next section. However, this can be a powerful strategy to further validate maps generated by ChIP-Seq or to assess bias imposed by chromatin fixation.

To date, two antibodies and one engineered protein have been shown to be compatible for endogenous expression followed by G4-mapping by ChIP. In 2016, the G4 parallel-specific D1 protein was expressed as an EGFP fusion antibody in the SiHa (carcinoma) cell line.^22^ Cells were transfected with a plasmid carrying the antibody sequence and incubated for 24 hours for transient expression of the antibody before cross-linking for ChIP-Seq. Chromatin fragments containing D1–G4 complexes were captured by protein A (pA) coated beads targeting the D1 antibody. This led to the detection of more than 8000 endogenous G4s, mainly located within gene bodies.^22^ The G4-specific artificial protein G4P developed from the G4-resolving helicase DHX36 has also proven to be compatible with stable expression for G4-mapping.^37^ G4P is composed of two DHX36 helical domains connected through a peptide linker of 18 amino acids. The addition of a nuclear localisation signal (NLS) and a 3xFLAG tag ensures its localisation within cell nuclei upon endogenous expression and renders it the smallest G4-binding protein probe with its ∼11 kDa size. G4P has been employed in ChIP-Seq following endogenous expression in several human cell lines.^37^ The number of G4s identified by G4P was variable according to the cell line studied and varied from 50 000 to 150 000 sites. This is 10-20 times higher than what was reported for ChIP-Seq using BG4 or SG4. This discrepancy could be due to differences in specificity of G4P and BG4 or in the different protocols used in the two ChIP-Seq approaches, using recombinant or endogenously expressed probes. Once expressed in living cells, G4P could compete against endogenous proteins for the same G4-binding site, thus recognising a higher number of G4s compared to recombinant BG4 or could induce the formation of some G4s that were not otherwise formed under endogenous conditions. In any case, it is hard to compare results obtained with different probes using different methods. The development of the nanobody SG4 allowed a first comparison between G4-maps obtained with extracted chromatin treated with BG4 and those obtained by endogenous expression of the probe. In essence, SG4 has been expressed endogenously as a protein fused with a nuclear localisation signal, a GFP to monitor the correct localisation in cell nuclei, and a 3xFLAG tag to immuno-precipitate the nanobody bound to G4s. The technique used to map G4s upon SG4 endogenous expression was CUT&Tag, a highly sensitive *in situ* method that requires less starting material compared to ChIP-Seq and that will be described in detail over the next section. SG4 expressed in HEK293T cells detected ∼8000 G4 sites, which is comparable to the number detected by recombinant SG4 in a standard ChIP-Seq protocol. This might suggest that the two approaches of using a recombinant and an endogenously expressed probes are similar and that the number of G4s not detected due to chromatin preparation biases is negligible. However, a direct comparison between the two approaches is not possible due to the difference in technique and in the cell lines used (ChIP-Seq in K562 and U2OS cells, CUT&Tag in HEK293T cells). Therefore, more data and evidence need to be gathered before making a final conclusion on the global differences in G4-detection that can be appreciated when expressing a G4-binding probe endogenously as opposed using it as a recombinant protein on extracted chromatin.

### *In situ* detection of G4 by CUT&Tag

Although the development of G4 ChIP-Seq has been a cornerstone to study G4-prevalence genome-wide, a significant limitation of this strategy is its inherently low resolution. Indeed, ChIP-Seq is based on the enrichment of G4-containing fragments over background DNA fragments, which are usually different in length as chromatin is randomly sheared during the sonication step. Therefore, this requires sequencing at a high depth (∼30 million reads) to detect G4-enriched fragments over the background. Moreover, ChIP steps like chromatin cross-linking, shearing and immunoprecipitation are technically laborious and may require time-consuming optimisation when new cell lines or organisms are investigated for the first time. Recently, the development of an *in situ* strategy has enabled a high-resolution map of G4s under native conditions, overcoming some of the limitations associated with ChIP-Seq. This method is known as CUT&Tag, or cleavage under targets and tagmentation,^34,38^ and is based on targeted cleavage of G4-containing DNA sites by a transposase enzyme (Tn5), as schematically illustrated in Fig. 4. In essence, this method is designed to process directly intact cells or nuclei (lightly fixed with 0.1% formaldehyde or native) that are held on magnetic beads covered in concavalin A (ConA). This is a protein from the lectin family that recognises glycoproteins and glycolipids present on cellular and nuclear membranes, which facilitates incubation of the samples with targeting probes and their subsequent processing (Fig. 4-(1)). Endogenous G4s are targeted *in situ* by a G4-specific antibody (*i.e.* BG4 or SG4) tagged with a 3xFLAG tag (Fig. 4-(2)). A second incubation with a rabbit anti-FLAG antibody is used to recruit a third IgG antibody (anti-rabbit) to the targeted G4-sites (Fig. 4-(3) and (4)). The anti-rabbit IgG antibody has two fundamental functions: firstly, it accumulates the Tn5 enzyme at the recognised G4-sites by direct interaction with the protein A (pA) fused to the transposase (pA-Tn5), enabling targeted digestion of the G4-containing regions. Secondly, it increases the sensitivity of G4-detection as multiple anti-rabbit antibody molecules are recruited at a single G4 site. The two incubation steps with the anti-FLAG and anti-rabbit IgGs are essential for the success of the protocol, since neither BG4 (scFV) nor SG4 (nanobody) are IgG types of antibodies that could be directly recognised by pA-Tn5. Successively, Tn5 enzymatic activity is activated by addition of Mg^2+^, which results in the cleavage of DNA sequences surrounding the labelled G4-sites (Fig. 4-(5)). At the same time, the Tn5 enzyme inserts next generation sequencing primer adaptors (tagmentation) as it cleaves the G4-containing site (Fig. 4-(5)). Tagmented DNA containing G4s is then amplified to generate next generation sequencing libraries (Fig. 4-(6)). G4 CUT&Tag has been used in a variety of cancer cell lines, including K562, U2OS, MCF7, 293T, HeLa, LM2, SW1271, and HaCaT,^34,39–41^ leading to the detection of ∼10 000–20 000 G4 sites, whose distribution is cell line-dependent and comparable to what observed by G4 ChIP. Moreover, a similar enrichment of G4s at promoters and enhancers was observed by CUT&Tag, further validating the observations previously reported using G4 ChIP-Seq.

The use of CUT&Tag has several technical advantages compared to ChIP-Seq. CUT&Tag is less time consuming and laborious compared to ChIP-Seq. By employing pA-Tn5 to cleave DNA surrounding G4 sites, CUT&Tag does not require any chromatin preparation, including chromatin extraction and sonication to generate DNA fragments for sequencing, and subsequent extra steps of quality control assessment. This also avoids any bias imposed by chromatin sonication and preparation. Moreover, heterochromatin is less prone to shearing upon sonication. This can increase the probability of generating different fragment size distributions according to the cell state during sample preparation in G4 ChIP, affecting immunoprecipitation and downstream sequencing, and introducing sample-specific biases that are avoided in CUT&Tag.^31,42^ Additionally, whilst ChIP-Seq requires chromatin fixation, CUT&Tag can be used on native samples, which are closer to the physiological chromatin status. From a more technical standpoint, it is worth highlighting that the library preparation for next generation sequencing is faster in CUT&Tag compared to ChIP, even though it is similar between the two techniques. This includes a step with a Tn5 that attaches sequencing adapters to DNA ends flanking G4s, followed by an amplification step (PCR), using barcoded primers. However, in CUT&Tag, DNA tagmentation occurs during the generation of DNA fragments, while, in ChIP-Seq, the tagmentation is an independent additional step performed after immunoprecipitation, making the CUT&Tag protocol leaner and less time consuming. Moreover, in CUT&Tag, DNA fragmentation by Tn5 digestion is targeted, so it occurs only at the sites interest. The digested fragments are consequently amplified by PCR, while the fraction of undigested DNA is not amplified. This enables a significant reduction of background fragments, leading to a higher signal-to-noise ratio compared to ChIP. For this reason, a lower sequencing depth (10 million reads) is required for CUT&Tag to obtain a high-resolution map of G4s. Conversely, in ChIP-Seq, the entire genome is fragmented by sonication, and non-G4 fragments which have not been removed during the wash steps are tagmented and retained in the sample, increasing the noise level.

With CUT&Tag, the higher resolution and the lower sequencing depth required enable the use of lower amounts of starting material needed to generate genome-wide maps of G4s. This is a great advantage when the sample source is limited, besides the obvious economic benefits. The development of CUT&Tag also allowed for G4-mapping at a single cell resolution. This could lead to the identification of individual G4 landscapes between different cell types within a small heterogenous population, for instance, in patient-derived cancer tissues. Indeed, standard ChIP-Seq or CUT&Tag provide a G4 map that reflects the average G4-distribution across multiple cells. Therefore, in a highly heterogenous population of cells, only G4-sites that are shared across multiple cells would be detected, whilst variations in the G4 profiles of individual cells would be lost in the noise.

As a proof of concept, single nuclei G4 CUT&Tag (snCUT&Tag) was used successfully to identify different cancer cell lines from an artificially mixed population of U2OS and MCF7, two different cancer types (bone and breast cancer, respectively), based on their individual G4 landscape.^40^ The rationale behind this was that the G4 profile would be cell line-specific, as observed in other genomics studies, allowing for the identification of distinct cell populations. In the longer term, this would open the possibility of exploiting G4s to study tumour heterogeneity, as these structures are enriched in cancer cells and the epigenetic plasticity typical of cancer is likely to affect G4-prevalence.^9^ Tumour tissues are composed of malignant cells at different stages of tumorigenesis and/or with different metastatic potential, and thus are genetically and epigenetically highly heterogeneous. For this reason, it has been hypothesised that, in the future, G4 genomic profiling could be used as a tool to discriminate different cell types in a heterogeneous population in patient tissues, and support cancer diagnosis.

## Chem-map: small molecule based G4 mapping

In 2023, small-molecule ligands were applied in genomic protocols using an *in situ* approach (Fig. 5) that is reminiscent of CUT&Tag.^43^ This technology, called Chem-map, involves the use of a biotinylated version of the G4-stabilising ligands PDS and PhenDC3 (PDS-btn and PhenDC3-btn) to label G4s throughout the genome in lightly fixed or native human cells/nuclei. The biotinylated ligands were then labelled with an anti-biotin rabbit IgG antibody (Fig. 5-(2)). A secondary IgG anti-rabbit antibody was incubated with a pA-Tn5 conjugate to form a complex that could be recruited to the G4 sites to cleave DNA and insert adapters for library generation, exactly as described for G4 CUT&Tag (Fig. 5(3)–(6)). Both PDS-btn and PhenDC-btn Chem-map could generate a high-resolution map of G4s that led to detection ∼10 000 G4 sites in the genome of K562 cells, which strongly overlaps with CUT&Tag data generated using the BG4 antibody (∼74% and ∼87% overlap, respectively). Thus, Chem-map provides a further validation of G4-maps across the human genome, strongly suggesting that the detected sites are independent of the type of probe used.

Chem-map is a versatile approach that can also be adapted to map the binding sites of other DNA targeting small molecules. For instance, besides G4 genomic profiling, Chem-map has been used to map binding sites of the BET proteins inhibitor JQ1, confirming strong overlap with previously reported BETs binding sites. Moreover, a map of JQ1's main target BRD4 was generated through CUT&Tag, revealing that more than 90% of JQ1 sites identified with Chem-map overlapped with BRD4 CUT&Tag. Additionally, this method has been used to identify the binding sites of the antitumour drug doxorubicin, which induces double strand breaks at gene promoters. Through Chem-map, it has been observed, for the first time, that biotinylated doxorubicin (Dox-btn1) binding sites are prevalently detected at promoters. Altogether, this evidence demonstrated that Chem-map is a robust approach to investigate the mode of action of small molecules targeting DNA or DNA binding proteins, including clinically approved drugs like doxorubicin.

This technique shares with CUT&Tag the advantage of requiring a limited number of cells, as previously discussed. Like CUT&Tag, Chem-map can be performed on native cells, although fixation is recommended when the ligand used to target a protein of interest that can compete with binding on the same DNA sites targeted by the probes. In this case, fixation would help preserve protein–DNA interactions. Currently, biotinylated modifications of G4 ligands such as PDS-btn and PhenDC3-btn used in Chem-map are not commercially available, rendering this method less accessible than others, as it requires in-house synthesis facilities. However, we anticipate that this might change in the future as the method gets used more widely and further developed, and potentially extended to the use of could be extended to other biotinylated G4-binding molecules that have been already used to pull down DNA and RNA G4s *in vitro*, such as TASQ.^44^

## G4access as a probe-free approach to G4 mapping

All the approaches to study the genomic distribution of G4s described in the previous sections relied on the use of targeting probes, either protein-based or small molecule ligands. Therefore, the detection of G4s is strongly dependent on the relative binding affinity and kinetics of the probe used, as well as on its selectivity for G4-binding. Consequently, some G4 sites might be recognised by more than one probe, while others might be uniquely identified or potentially missed depending on the type of probe used. Moreover, G-rich sequences with lower propensity of forming G4s may be induced to fold into these structures by the presence of the probes themselves, potentially introducing false positives.

G4access has been recently presented as an approach to overcome these limitations and further validate the detection of G4 forming sequences (G4FS) in open chromatin regions with a probe- and crosslinking-independent approach.^45^ As reported in Fig. 6, in G4access, native cell nuclei are incubated with micrococcal nuclease (MNase), which preferentially cuts before G-stretches (Fig. 6-(2)). Then, the DNA fragments are extracted and separated by gel electrophoresis (Fig. 6-(4)). This is performed to select subnucleosomal DNA fragments where most of the G4s are expected to form. The electrophoretic gel is cut, and small beads of gel are generated through poking with a 0.45 μm needle (Fig. 6-(5)). After DNA extraction and purification, sequencing libraries are generated and sequenced (Fig. 6-(6)). By developing this strategy, Esnault *et al.* reported ∼44 000 genomic G4s in K562 cells, ∼11 000 in Raji cells and ∼12 000 in HaCaT cells, with 4743 G4s consistently shared across the three cell lines. The authors validated *in vitro* the formation of G4 structures for more than 500 of genomic G4s identified across the three cell lines, confirming that the 80% of the sequences analysed have the potential to fold into G4s.

G4access is a valid orthogonal approach to confirm the results obtained using probe-based techniques to study the genomic location of G4s, especially for G4 structures located in nucleosome-free regions. G4access has the great advantage of being free from biases introduced either by G4-probes or by chromatin cross-linking, allowing the identification of G4s regardless of the presence of G4-binding proteins potentially hindering probe binding. On the other hand, this approach is purely based on the enzymatic activity of MNase, which preferentially cleaves A- and T-rich regions and genomic sites upstream of any G-stretches regardless of these sequences being folded into a G4-structure or not. The method, in fact, requires *in vitro* validation of G4-formation for any of the sequences identified, which is performed on synthetic oligonucleotides and does not necessarily reflect the status of G4-folding in chromatin. Whilst G4access offers the advantage of being a probe-free method, it comes with the strong limitation of only indirectly detecting G4s in chromatin by relying on enzymatic digestion of A–T rich DNA stretches, leading to a biased enrichment of G-rich sequences irrespectively of whether they contain or not a G4-structure in the first place. *In vitro* validation of G4-formation identified with G4access cannot be used as a reliable proxy for G4-folding in chromatinised DNA. Therefore, we strongly recommend orthogonal validation of G4-maps obtained by G4access with probe-based methods like CUT&Tag or ChIP-Seq.

## Conclusions and future challenges

Several chromatin features are essential for the regulation of cellular processes, such as transcription and replication. Over the past decade, G4-structures have been gaining traction as alternative chromatin features that can play a role in orchestrating transcriptional and epigenetic regulation. To fully unravel the relevance of G4s in chromatin organisation and epigenetic regulation, there is a need to generate detailed and reliable G4 maps within the genome. To achieve this, standard genomics methods (*i.e.* ChIP-Seq and CUT&Tag) have been adapted over the past decade to allow for G4-mapping by using G4-specific antibodies. Although the use of G4-selective antibodies is often described as a limitation, it is highly reminiscent of standard genomics methods used for mapping DNA binding proteins like transcription factors, or other nucleic acids structures like R-loops.^34,46^ One attempt to directly detect G4s in the human genome through immunoprecipitation was reported in 2013 by employing the G4-binding antibody hf2 on purified DNA fragments from MCF7 cells.^47^ However, like G4-Seq, this approach excludes the chromatin status that may regulate the formation of G4s, as genomic DNA is extracted and treated with proteases to eliminate chromatin-associated proteins. Before the generation of antibodies compatible with ChIP-Seq protocols, the genomic location of G4s has been studied more indirectly by mapping of G4-binding proteins or proteins associated with G4-stablising phenotypes. Some examples are offered by Law *et al.* (2010) and Gray *et al.* (2014), who performed ChIP-Seq targeting the G4-binding proteins ATRX and XPB/XPD, respectively.^48,49^ Similarly, the DNA damage marker γH2AX was mapped by ChIP-Seq upon treatment with the G4 stabiliser PDS to infer G4 locations from DNA damage sites elicited by PDS treatment.^21^ The development of G4 ChIP-Seq using the G4-specific antibody BG4 by Hänsel-Hertsch *et al.* (2016) revolutionised the study of G4-biology, allowing the direct mapping of G4s within functional genomic regions in a chromatin context, revealing cell to cell variations.^9^ Since the generation of the first G4 map in human chromatin, there have been many attempts to study G4-biology under conditions as close as possible to the native state of the cell. For example, the use of probes that can be stably expressed in nuclei or using approaches that do not require chromatin fixation (CUT&Tag). The development of CUT&Tag facilitated the generation of a genomic G4 map at high resolution, which also enables further studies at a single cell resolution. This technique has also been optimised for the use of G4-stabilising ligands rather than antibodies (Chem-map), offering a robust validation of the results obtained with protein-based probes.

Significant differences in number and location have been observed in different cell lines regardless of the method employed and the probe used. Similarly, a good overlap of G4-maps obtained with different methods have been reported, which collectively suggests the potential for a functional role of G4s in the regulation of chromatin architecture and epigenetic plasticity. Now, with further development and refinement of genomics methods to map G4s, the scientific community has the tools to address the mechanistic role of G4-formation and its impact in epigenetic reprogramming and chromatin architecture. Indeed, the production of genome-wide G4 maps has been essential to further our understanding on G4-biology and to validate formation of these structures in living cells. Further development of these methods could help to identify changes in G4 landscape that might drive epigenetic plasticity in disease states like cancer. Moreover, whilst the methods described in this article are optimised for human cells, they could be extended to other model organisms to study their biology from a fresh perspective.

On the other hand, all the genomics methods developed to date provide a mono-dimensional map of G4s throughout the genome and do not consider the role of G4s in establishing long-range chromatin interactions that occur *in vivo*. DNA looping is a well-known phenomenon happening in chromosomes, by which chromatin folds in loops bringing in close physical proximity two distal DNA sequences and can strongly affect transcriptional regulation. This process is essential in the epigenetic control of gene expression by reducing the distance between genes and regulatory regions called enhancers, which are sequences that recruit additional factors to regulate the level of transcription at distal genes. It has been already observed that G4s are recognised by proteins involved in DNA looping, for instance YY1 and CTCF.^11,12^ BG4 ChIP sites highly overlap with both YY1 and CTCF ChIP sites as well as RNA polymerase II (RNA pol II).^50^ Additionally, the overlap between G4s and YY1 and RNA pol II sites decreases upon treatment of G4-stabilising ligands, supporting the idea that G4s may be directly involved in the establishment of 3D chromatin architecture. However, current G4-mapping methods cannot be used to investigate the formation of inter-molecular G4-structures that might orchestrate such DNA looping, which are also observed to contribute to the formation of condensates.^51,52^ This is becoming of more importance, given that the chromatin remodeller CSB has been shown to selectively bind intermolecular G4-structures,^53^ further suggesting that G4s can have a functional role in DNA looping. To generate direct evidence for the presence and contribution of inter-molecular G4s at looping sites, the development of novel genomic strategies that allow to detect multi-molecular G4s is required. We anticipate that such methods are likely to be reminiscent of the Hi-C approaches currently used to study distal genomic interactions.

**Fig. 7:**
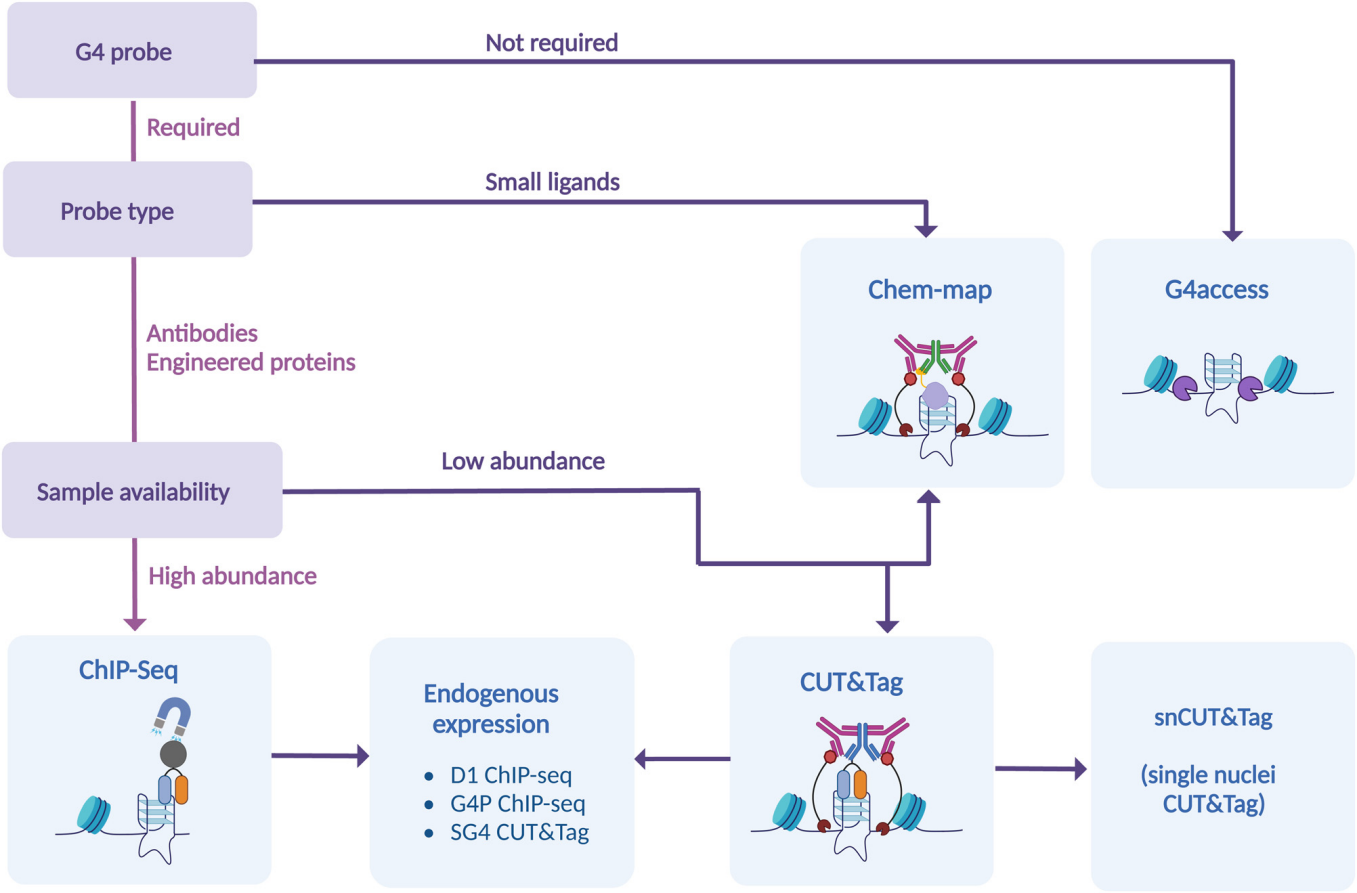
Current genomic approaches to G4 mapping. Genome-wide G4 mapping techniques are divided according to their main features. These include employment of a probe, type of probe used, the requirement of a fixative to retain chromatin structure, or the possibility of maintaining native conditions.

Overall, we now have a range of genomic methods to study in detail, and with confidence, intra-molecular G4-distribution at a genome-wide scale. In this critical review, we have highlighted the strengths and the weaknesses of each of these methods, offering a guide on selecting the best strategy to accommodate experimental requirements (Fig. 7). Additional methods that take into account long-range G-G base pairing and allow for selective detection of inter-molecular G4s in chromatin are needed to generate a comprehensive understanding on the epigenetic function of G4-structures. Nevertheless, it is becoming increasingly apparent that the potential of G4s to modulate epigenetics and transcription is higher than perhaps initially thought.

## Author contributions

Conceptualisation: M. D. A., S. G.; writing: M. D. A, S. G. and G. F. with support from L. R.; figures: G. F. and S. G. All authors have read and agreed to the published version of the manuscript.

## Conflicts of interest

There are no conflicts to declare.

## Supplementary Material
